# From Models to Measurements: Comparing Downed Dead Wood Carbon Stock Estimates in the U.S. Forest Inventory

**DOI:** 10.1371/journal.pone.0059949

**Published:** 2013-03-27

**Authors:** Grant M. Domke, Christopher W. Woodall, Brian F. Walters, James E. Smith

**Affiliations:** 1 USDA Forest Service, Northern Research Station, St. Paul, Minnesota, United States of America; 2 USDA Forest Service, Northern Research Station, Durham, New Hampshire, United States of America; DOE Pacific Northwest National Laboratory, United States of America

## Abstract

The inventory and monitoring of coarse woody debris (CWD) carbon (C) stocks is an essential component of any comprehensive National Greenhouse Gas Inventory (NGHGI). Due to the expense and difficulty associated with conducting field inventories of CWD pools, CWD C stocks are often modeled as a function of more commonly measured stand attributes such as live tree C density. In order to assess potential benefits of adopting a field-based inventory of CWD C stocks in lieu of the current model-based approach, a national inventory of downed dead wood C across the U.S. was compared to estimates calculated from models associated with the U.S.’s NGHGI and used in the USDA Forest Service, Forest Inventory and Analysis program. The model-based population estimate of C stocks for CWD (i.e., pieces and slash piles) in the conterminous U.S. was 9 percent (145.1 Tg) greater than the field-based estimate. The relatively small absolute difference was driven by contrasting results for each CWD component. The model-based population estimate of C stocks from CWD pieces was 17 percent (230.3 Tg) greater than the field-based estimate, while the model-based estimate of C stocks from CWD slash piles was 27 percent (85.2 Tg) smaller than the field-based estimate. In general, models overestimated the C density per-unit-area from slash piles early in stand development and underestimated the C density from CWD pieces in young stands. This resulted in significant differences in CWD C stocks by region and ownership. The disparity in estimates across spatial scales illustrates the complexity in estimating CWD C in a NGHGI. Based on the results of this study, it is suggested that the U.S. adopt field-based estimates of CWD C stocks as a component of its NGHGI to both reduce the uncertainty within the inventory and improve the sensitivity to potential management and climate change events.

## Introduction

The ecological importance of trees in forest ecosystems extends well beyond their biological life in both space and time [1], [2]. When trees fall or shed components (e.g., branches), downed and dead woody material (DWM) is created, providing critical substrate for the establishment of vegetation, habitat for wildlife species, and nutrients for a variety of forest ecosystem functions [3]–[6]. The benefits of DWM in forests and, indirectly, to society can be at odds with the fact that DWM may also hinder forest management activities, provide habitat for forest pests, and increase wildfire risk [1], [7]. Quantifying DWM in natural and managed forest ecosystems has been critical to understanding how disturbance (natural and anthropogenic) and other biotic/abiotic factors influence DWM dynamics. Much of the research on DWM is regionally specific and has been conducted at varying scales to assess fuel loads [8], [9], wildlife habitat [1], [3], [10], [11], or carbon (C) [12]–[14]. The results from such studies have been used to develop relationships with other forest ecosystem attributes (e.g., live and/or standing dead tree biomass/C) to approximate DWM biomass and C stocks at multiple spatial and temporal scales [13], [15], [16], [17].

Forest C stocks in the U.S. are estimated using data from the national forest inventory (NFI) conducted by the USDA Forest Service, Forest Inventory and Analysis (FIA) program. Estimates of live and standing dead tree C stocks are based on biomass estimates obtained from inventory tree data [18], [19]. Estimates of DWM C used in past U.S. National Greenhouse Gas Inventories (NGHGI) were calculated using models with geographic area, forest type, and live tree C density as independent variables [20]. The FIA program has measured DWM attributes as part of the strategic NFI since 2001 [21]. Estimators have been developed to compute DWM volume, biomass, and C [22]; however, since DWM inventories have been initiated by individual states at varying times over the last decade, before now there has been insufficient data to generate consistent national population estimates that meet the precision standards established by the FIA program [23]. In an effort to begin incorporating field-based estimates of DWM C stock estimates into the NGHGI report, the FIA program compiled all DWM C attributes from 2002–2010 and adjusted state-level population estimates of DWM in the current NGHGI to reflect field-based population estimates [24]. The latest compilation also provides an opportunity to examine the models used to estimate plot- and population-level estimates of DWM C stocks and compare model- and field-based estimates. As DWM models have been used for over a decade [22] to inform the U.S.’s NGHGI, the transition to field-based DWM C density estimates should be evaluated to inform policy makers and stakeholders from the entity (i.e., C projects – [25]) to international level (e.g., *Good Practice Guidance for Land Use, Land-Use Change and Forestry*, Intergovernmental Panel on Climate Change, IPCC [26]). Beyond the borders of the U.S., the potential benefits of any nation adopting a robust tier three approach [26] to DWM C pool monitoring (i.e., field-based inventories of DWM), as opposed to other tiers that rely solely on models, has never been evaluated. Given the costs associated with field-based C inventories and difficulties in achieving the statistical power to detect C flux [27], the evaluation of DWM field inventory efficacy is paramount to monitoring forest ecosystems in the context of global change.

The goal of this study is to examine the effect of incorporating field-based estimates of DWM C stocks into the U.S.’s NGHGI report by comparing differences in model- and field-based estimates of DWM C stocks within the FIA program. The specific objectives of the analysis are to: 1) assess the performance of DWM C stock models by region, ownership, and forest type in the FIA database, 2) compare model- and field-based estimates of DWM C stocks by region and ownership, and 3) describe recent changes in the estimation of DWM C stocks in the NGHGI report and suggest directions for future research.

## Methods

### Downed and Dead Woody Material Definitions

The FIA program defines DWM in forest ecosystems as detrital components inclusive of fine woody debris (FWD) and coarse woody debris (CWD), including CWD amassed in piles resulting from forest management activities or disturbance events [20]. This study focuses specifically on downed dead wood as it is defined in the NGHGI as pieces, or portions of pieces, of downed dead wood (minimum small-end diameter ≥7.62 cm at the point of intersection with a sampling transect and a length ≥0.91 m) including an additional component of downed dead wood amassed in piles [20]. Fine woody debris (small-end diameter <7.62 cm), although inventoried by the FIA program, are not included in this analysis as the forest floor C pool is currently defined as including this material in the NGHGI [20]. It is hoped that FIA’s field-based inventory of the forest floor pool [28] can someday better align with populations defined by the NGHGI (e.g., large FWD as a component of the DWM pool) such that field-based estimates can be included in the NGHGI reporting. Coarse woody debris as defined in Woodall and Monleon [22] must be separate from a standing dead tree and have a lean angle greater than 45 degrees from vertical. Coarse woody debris amassed in piles (i.e., slash piles) is defined as spatial assemblages of downed dead wood that can be delineated in terms of size and piece density (i.e., packing ratio; [29]). When reporting DWM in the U.S.’s NGHGI, individual CWD pieces and slash piles are combined [20]. For the purposes of this study and in alignment with the NGHGI, CWD will refer to both individual pieces and slash piles except when explicitly noted.

### Plot-based Sampling Protocol

The FIA program maintains a three-phase inventory program, where Phase 1 is designed to reduce variance through stratification using satellite imagery to assign Phase 2 plots to strata [23]. Site and tree attributes are measured at regular intervals on Phase 2 plots that contain a forest land use. Phase 2 plots are quasi-systematically distributed every 2,428 ha across the U.S. Coarse woody debris attributes are typically measured on every 16^th^ Phase 2 plot (38,866 ha) as part of the Phase 3 sample. In a few regions, states (e.g., west Texas, Oregon, Washington) have elected to sample CWD at the same intensity as Phase 2. Phase 2 and 3 plots are comprised of four 7.32-m fixed-radius subplots spaced 36.6 m apart in a triangular arrangement with one subplot in the center [23]. Coarse woody debris is sampled on transects radiating from each Phase 3 subplot center (at angles 30, 150 and 270 degrees, respectively). Each subplot has three 7.32 m transects, totaling 87.8 m for a fully forested inventory plot [22]. Data collection involves recording every CWD piece intersected by a transect, and measuring transect diameter, length, small-end diameter, large-end diameter, decay class, and species. Transect diameter is the diameter of a downed woody piece at the point of intersection with a sampling transect. Decay class is a subjective determination of the amount of decay present in an individual log. Decay class 1 is the equivalent of a freshly fallen log (the least decay), while decay class 5 is extremely decayed [30]. Fallen logs are identified to species using species-specific bark, branching, bud, and wood composition attributes (excluding decay class 5). Coarse woody debris found in piles – regardless of cause – with the pile center coinciding with a subplot is sampled using pile protocols rather than sampling transects. Field crews assign the pile a shape category (i.e., circular or rectangular), measure its dimensions, and visually assess the pile in terms of the density of CWD within the defined shape [22], [29].

### Study Regions and Data

**Figure 1:**
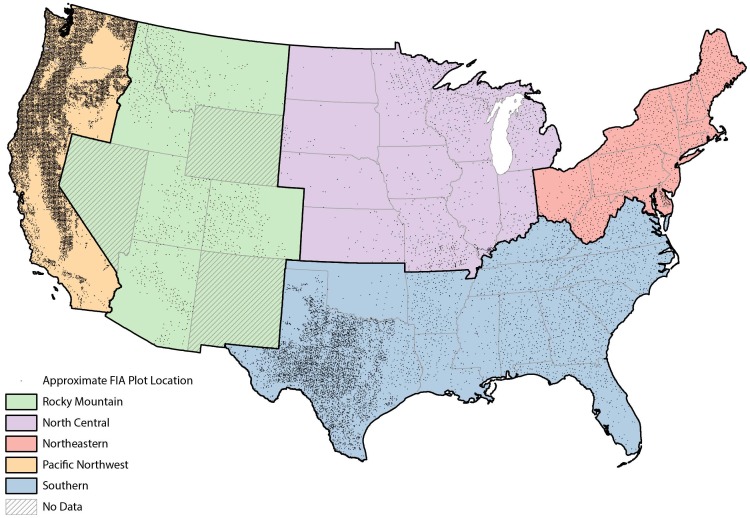
Study regions and approximate Forest Inventory and Analysis plot locations. Coarse woody debris is typically measured on Phase 3 plots in the USDA Forest Service, Forest Inventory and Analysis program. In the Pacific Northwest (CA, OR, and WA) region, and west Texas, coarse woody debris was measured on all Phase 2 plots substantially increasing the observations (*n* = 16,323 and *n* = 5,403, respectively) in those regions.

Field data for this study were taken entirely from the FIA database [30], [31], sampled from 2002–2010 in the 48 conterminous states of the U.S. for a total of 22,641 unique inventory plots (Figure 1). The data were organized by region and ownership (i.e., public and private) to account for potential differences in forest land management (Table 1). As CWD inventories were initiated at varying times from 2002–2010, sample intensities vary by state. In addition, states have the opportunity to increase the sample intensity of both Phase 2 and Phase 3 plots. Furthermore, states also have the opportunity to increase the size of the fixed-area sample plots from 7.32 m to 17.95 m, with a commensurate increase in the length of CWD transects. Changes in both fixed-area subplot size and CWD sampling transects were incorporated into estimation procedures to allow seamless comparison across the entire U.S.

**Table 1:** Summary of plot-level attributes (mean and standard deviation) for each region in the study.

Region	Number of observations	Mean age (years)	Max age (years)	Site index (m)^1^		Basal area (m^2^)	
**Public forest land**							
Northeast	243	67	169	19.1	(5.8)	27.2	(14.3)
North Central	463	64	222	18.0	(4.8)	23.4	(29.8)
South	644	46	160	24.8	(6.2)	17.4	(15.3)
Intermountain West	644	110	460	12.7	(4.6)	21.8	(17.2)
Pacific Northwest	10075	109	900	21.6	(7.7)	32.1	(29.1)
**Private forest land**							
Northeast	1001	59	139	18.9	(5.5)	24.7	(13.1)
North Central	840	57	200	18.9	(5.0)	21.9	(14.9)
South	7449	36	550	25.2	(5.9)	12.6	(32.1)
Intermountain West	244	86	367	12.8	(4.6)	16.5	(14.4)
Pacific Northwest	6248	56	600	25.8	(7.9)	26.1	(99.6)

1 Only site index estimates with a base age 50 years were used in this estimate.

Plots were summarized for each region and ownership by mean and maximum age, site index (base age 50 years), and basal area (Table 1). Estimates of C density in live and standing dead trees were also summarized by region and ownership for the CWD plots used in the study. Gross volume of live and standing dead trees was estimated from tree attributes (e.g., diameter at breast height, tree height) using species- and region-specific models [18]. Gross volume was then adjusted to account for rough, rotten, or missing material and converted to biomass using specific gravity of wood and bark by species and the weight of water. Estimates of standing dead tree biomass were further adjusted to account for decay and/or missing tree components [19] and both live and standing dead tree biomass was converted to C following Woodall et al. [18]. The estimated C content in individual trees was then expanded to a per-unit-area basis (Mg•ha^−1^) using expansion factors [23].

### Field-based Estimates of C density in CWD

Field-based estimates of C in CWD were determined through application of estimators detailed in Woodall and Monleon [22]. Briefly, volume was computed for every CWD piece, then used in an estimator to estimate per-unit-area volume [22], [32] on each plot in the FIA database [23], [31]. Volume was converted into biomass through the use of density and decay reduction factors (i.e., reducing wood density through classes of decay) and then converted to C based on individual pieces’ species and decay class [33]. For a complete description of the CWD estimator, including estimates associated with individual FIA plots and domains of interest (e.g., states), please refer to Woodall and Monleon [22].

### Model-based Estimates of C density in CWD

Until recently, there has not been sufficient data from the subset of FIA plots where CWD attributes have been measured to compute population estimates of CWD C stocks to meet NGHGI reporting requirements [15], [20]. Instead, estimates have been computed – largely based on the relationship between CWD and live tree C density – using simulations from the Forest Carbon Budget Model (FORCARB2; [34]) and applied to the plot level [20]. These estimates, hereafter referred to as model-based estimates of C in CWD, continue to be used in the FIA database – expressed on a per-unit-area basis – and were used in conjunction with the recently compiled field-based estimates of C in CWD to compute the CWD C estimates for the 2013 NGHGI report [24]. The model-based procedure used to estimate per-unit-area and population-level C estimates of CWD in this study and the 2013 U.S. NGHGI report is as follows:
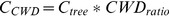
(1)Where *C_CWD_* = C density (Mg•ha^−1^) in CWD not including logging residues (i.e., slash piles), *C_tree_* = plot-level C density (Mg•ha^−1^) in above and belowground live trees, and *CWD_ratio_* = region- and forest type-specific ratios developed from FORCARB2 and found in Table A-225 of the 2013 NGHGI report [24]. An additional term is included in the plot-level estimate to account for logging residue [16], [20],
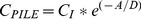
(2)where C*C_PILE_* = C density of wood and bark in slash piles, C*_I_* = regional mean C density (Mg•ha^−1^) by hardwood or softwood group at age zero (Table A-226, [24]), e = base of the natural logarithm (2.718), A = age in years, and D = first order decay coefficient by region and hardwood or softwood group (Table A-226, [24]). The sum of C*_CWD_* and C*_PILE_* are used to produce per-unit-area estimates of C density in CWD in the NGHGI report.

### Field Adjusted NGHGI Estimates

As there is a need to both “downscale” CWD C estimates from the NGHGI to individual plots and “back cast” contemporary estimates to the 1990 baseline year, an additional adjustment term was used in the NGHGI report to align the field- and model-based estimates,
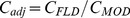
(3)where *C_adj_* = ratio of field-based C density in CWD to model-based C density in CWD, *C_FLD_* = field-based state-level population estimates of C density (Tg) in CWD and *C_MOD_* = model-based (FORCARB2; [34]) state-level population estimates of C density (Tg) in CWD. Field-adjusted C density estimates are then,
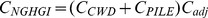
(4)where C*_NGHGI_* = field-adjusted estimate of state-level population C density in downed dead wood in the NGHGI report [23].

### Data Analysis

The quality of fit between the model- and field-based estimates were examined using two approaches. First, region- and forest type-specific model estimates were compared with field observations using a technique known as modeling efficiency [35]:
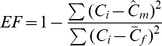
(5)where 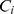 = field-based estimate of C density in CWD, 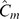 = model-based estimate of C density in CWD, and 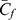 = mean field-based estimate of C density in CWD by region and forest type. This approach provides an index of model performance on a relative scale where 1 indicates a ‘perfect’ fit, 0 suggests the model is no better than the mean, and negative values indicate a poor model fit.

The second technique for assessing model-based estimates to field data required: 1) ordering 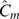, and 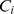 with respect to 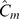, 2) grouping estimates by 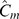 to produce at least 100 groups with group sizes not exceeding 200, 3) for each group, *g*, the means (and associated standard deviations) were calculated,
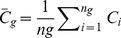
(6)

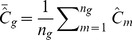
(7)where 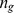 was the number of estimates in the g^th^ group, 4) the group means, 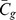
(field-based) and 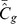
(model-based) were plotted against one another, and 5) a 1∶1 line was constructed to assess the quality of fit of the model to the field data. If the model fits the data, the data points should lie along the 1∶1 line with intercept 0 and slope 1.

The CWD data were heavily right-skewed so a bootstrapping technique was used to draw samples (*n* = 1000) of the data and the quantiles of the empirical distribution of the C density statistic from the drawn samples were plotted against the normal distribution to ensure that the sample for each region and ownership was sufficiently large to invoke the Central Limit Theorem for subsequent analyses.

To assess whether the per-unit-area estimates produced by the model and field data can be regarded as functionally equivalent, a test of equivalence [36] was used to compare differences. This approach reverses the typical “burden of proof” of statistical hypothesis testing, where “no difference” is treated as the null hypothesis and the data must demonstrate that an actual difference exists. Rather, this method assumes the estimates are not equivalent unless the data demonstrate that the estimates are similar to within a predefined tolerance. Regression-based approaches are regarded as the most powerful tests of equivalence [37]. However, we opted to use two-one-sided t-tests (TOST) due to the large number of model- and field-based estimates of CWD C density at or approaching 0 Mg•ha^−1^ [36]. Furthermore, we chose to test the mean and median of the ratios between field- and model-based estimates due to the large number of values at or near zero. A ratio with associated confidence limits around 1 would indicate the model- and field-based estimates are equivalent. Specifically, we defined “broad” (±0.25, confidence limits (CL) between 0.75–1.25) and “narrow” (±0.10, CL between 0.90–1.10) regions of indifference (i.e., tolerance interval) for the TOST. Under the TOST approach, when using a nominal α = 0.05, equivalence is demonstrated if the 90 percent CL of the mean or median of the ratio between estimates fall within the tolerance interval. Confidence intervals for the mean were calculated following standard parametric procedures and the CL for the median were calculated using a nonparametric Wilcoxon approach [38]. All analyses were conducted using R 2.13.1 statistical software with the boot and equivalence packages [39].

## Results

**Figure 2:**
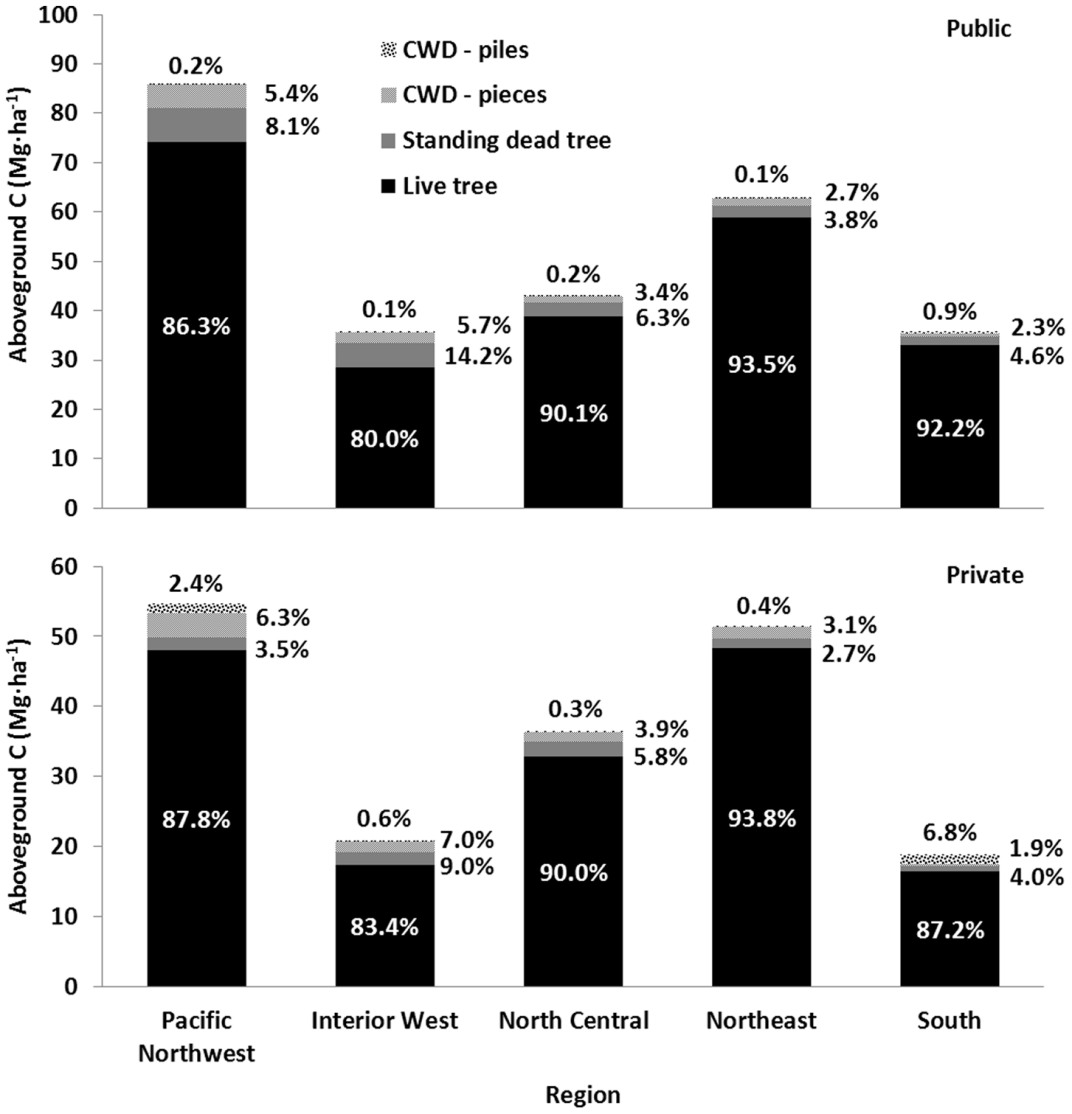
Estimated aboveground forest carbon density by ownership and study region. Field-based measurements of live tree, standing dead tree, and coarse woody debris (delineated by individual CWD pieces and CWD slash piles) were used to calculate carbon density (i.e., per-unit-area) estimates.

The differences in stand attributes between public and private ownership across the five regions reflect potential differences in forest management intensity and utilization (e.g., lower stand age on private lands; Table 1). The distribution of plots on public and private forest lands across the five regions also reflect the general land ownership patterns in the conterminous U.S. – the majority of plots in the east were on private forest land and a plurality of plots in the west were on public forest land. Total aboveground forest C (standing live/dead trees and field-based CWD) densities (Mg•ha^−1^) were higher, on average, on public land in all five regions in the study. The Pacific Northwest (PNW) region had the highest C density (i.e., per-unit-area), on average, with nearly 85.9±92.4 Mg•ha^−1^ (mean ± SD) on public land and 54.6±64.3 Mg•ha^−1^ on private forest land (Figure 2). The Southern (SO) region had the lowest aboveground C density estimates with 35.8±39.1 Mg•ha^−1^ on public land and 18.8±30.4 Mg•ha^−1^ on private forest land. The proportion of aboveground C density differed slightly among the three C pools included in the analysis across the different regions in the study. Coarse woody debris represented a larger proportion of total aboveground C stocks on private forest lands in all regions, with the highest proportions in the PNW and SO. Slash piles within the CWD pool were a relatively minor component on public forest lands with the exception of the SO region, where more than 27 percent (0.31±0.11 Mg•ha^−1^) of the C in the pool was from slash piles (Figure 2). Piles on private forest lands represented a much larger proportion of the CWD C density, accounting for nearly 78 percent (1.28±0.23 Mg•ha^−1^) of the total CWD C density in the SO region and 27 percent in the PNW (1.31±0.41 Mg•ha^−1^). Conversely, standing dead tree C stocks represented a larger proportion of aboveground forest C on public than private forest land across all regions.

**Figure 3:**
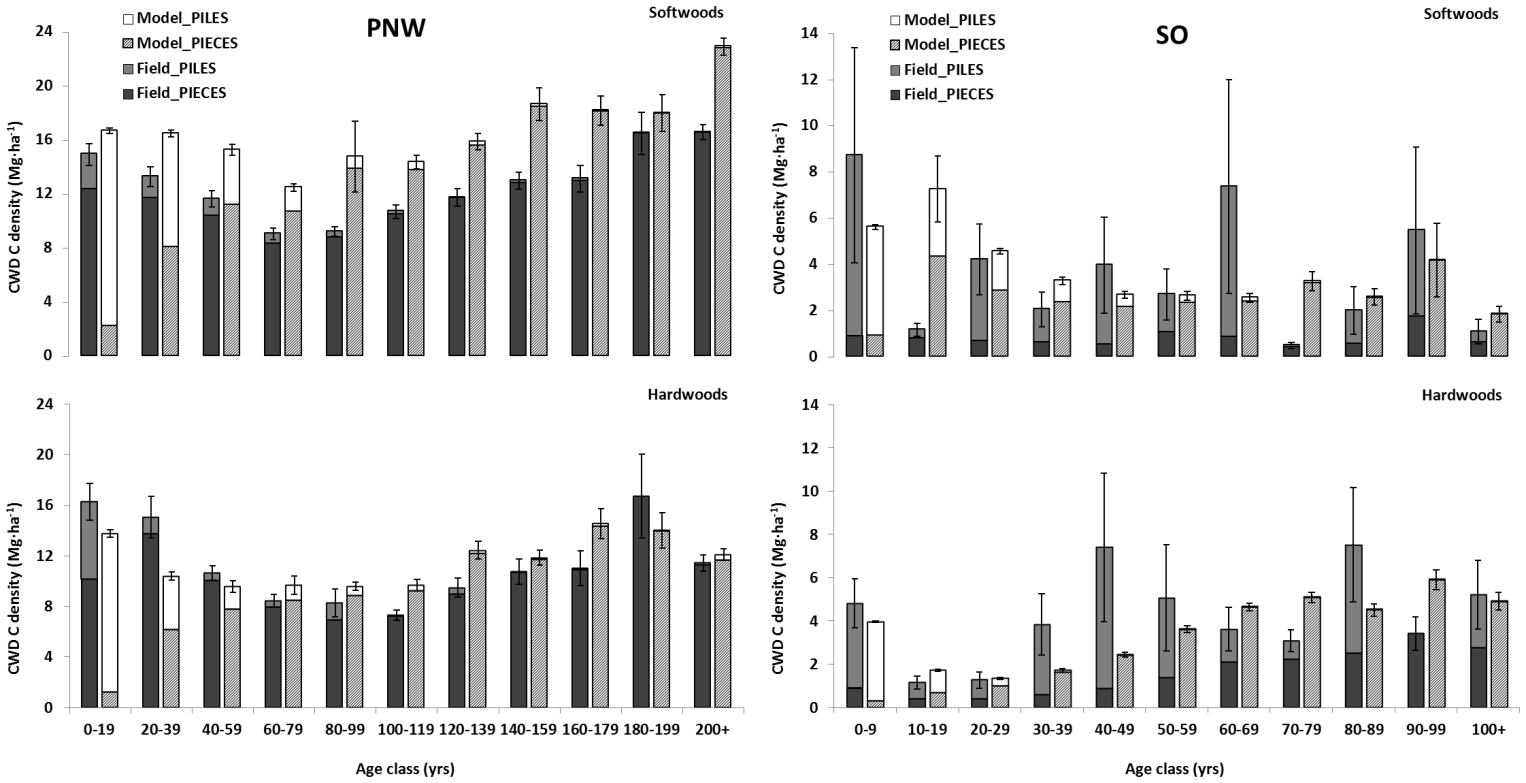
Estimated coarse woody debris carbon density by stand age, region, and broad forest type group. Model-based estimates of slash piles (i.e., logging residues) are calculated by region, broad forest type group (softwood/hardwood), and stand age in the U.S. National Greenhouse Gas Inventory Report [20]. Field- and model-based estimates (with standard errors) of coarse woody debris carbon density by component (i.e., pieces and slash piles) were compared for all observations in the Pacific Northwest (PNW; *n* = 16,323) and South (SO; *n* = 8,093) regions.

Model-based estimates of CWD slash piles (i.e., logging residue) were estimated by region, hardwood and softwood forest type group, and stand age in the NGHGI [20]. To evaluate the contribution of each CWD component (i.e., pieces and slash piles) to the total C density (per-unit-area) of CWD, model- and field-based estimates of CWD C from pieces and slash piles were compared by region, broad forest type group, and stand age class. In general, for all forest types and regions, the model overestimated CWD C from slash piles early in stand development (i.e., 0–40 years) and underestimated the contribution of C from CWD pieces in young stands (Figure 3). In the PNW region, where piles were a major component of aboveground C stocks, model-based estimates of CWD C per-unit-area in slash piles were more than 80 percent greater than field-based estimates in the first two age classes (11.86 Mg•ha^−1^ and 6.84 Mg•ha^−1^, respectively). This trend continued, to a lesser extent, into the older age classes for both hardwood and softwood forest types in the PNW. Model estimates of C in CWD pieces, on the other hand, were substantially smaller than field-based estimates early in stand development and then in the 40–59 year age class, models began overestimating C density of CWD pieces (Figure 3). In the SO region, model-based estimates of C in CWD pieces were similar to field-based estimates in the first 10 years of stand development but, thereafter, model-based estimates were substantially greater that field-based estimates for both hardwood and softwood forest types. The field-based estimates of C by CWD component suggested the majority of C was in slash piles across all but a few age classes for both hardwood and softwood forest types (Figure 3). Unlike other regions, model-based estimates of C in slash piles in the SO were substantially smaller than field-based estimates in most age classes.

**Figure 4:**
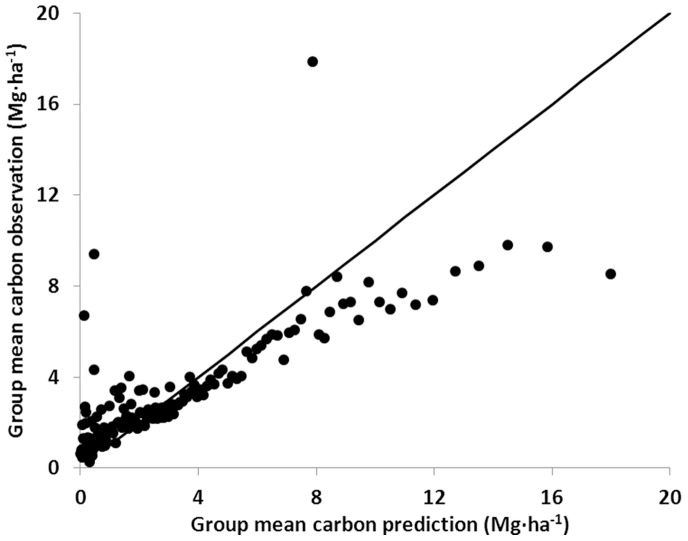
Group means of field- and model-based estimates of coarse woody debris carbon density. Individual (per-unit-area) estimates of field- (observation) and model-based (prediction) coarse woody debris carbon were ordered and grouped with respect to the model-based estimates into at least 100 groups with group sizes not exceeding 200. Group means were calculated and plotted against one another and a 1∶1 line was constructed to assess the quality of fit of the model to the field data. If the models fit the field-based data perfectly, the data points should lie along the 1∶1 line with intercept 0 and slope 1. Note that extreme outliers were omitted for graphing purposes.

The fits of region- and forest type-specific CWD C density models to field data produced EF values ranging from highly negative for forest types not well represented in certain regions (e.g., exotic softwoods in the Northeast [NE] and exotic hardwoods in the PNW) to 0.48 for the Douglas-fir forest type in the Intermountain West (IW) region (Table 2). Other forest type models that performed better than the mean included Western larch and Other western softwoods types at 0.14 and 0.12, respectively, in the IW regions. The large number of negative EF values suggest poor model fits to the field-based CWD C density estimates throughout most forest types and regions. The graph of group field-based means versus group model estimates (Figure 4) indicates a general lack of fit, with the models underestimating CWD C density on plots with a small amount of observed CWD C and overestimating CWD C density on plots with a large amount of observed CWD C density. Not surprisingly, there is also a substantial amount of spread in the field-based estimates of CWD C density relative to the smoothed estimates from the model-based approach.

**Table 2:** Quality of fit metrics (EF) for model-based versus field-based estimates of coarse woody debris carbon by region and forest type.

	Intermountain	North		Pacific	
Forest type	West	Central	Northeast	Northwest	South
White/red/jack pine	–	−0.30	−0.61	–	−75.00
Spruce/fir	–	−0.47	−0.31	–	–
Longleaf/slash pine	–	–	–	–	−1.09
Loblolly/shortleaf pine	–	−6.88	−2.69	–	−0.19
Other eastern softwoods	–	−35.76	–	–	−0.01
Pinyon/juniper	−0.02	–	–	−0.34	0.00
Douglas-fir	0.48	–	–	−0.30	–
Ponderosa pine	−0.11	0.18	–	−0.83	–
Western white pine	–	–	–	−0.28	–
Fir/spruce/mountain hemlock	−0.13	–	–	−0.21	–
Lodgepole pine	−0.31	–	–	−0.19	–
Hemlock/sitka spruce	−0.35	–	–	−0.59	–
Western larch	0.14	–	–	−0.04	–
Redwood	–	–	–	−0.10	–
Other western softwoods	0.12	–	–	−0.18	–
California mixed conifer	–	–	–	−0.69	–
Exotic softwoods	–	−0.39	−332.03	–	–
Oak/pine	–	−0.10	−2.72	–	−0.04
Oak/hickory	–	−2.20	−0.34	–	−0.01
Oak/gum/cypress	–	–	−1.67	–	−0.59
Elm/ash/cottonwood	−0.90	−1.08	−5.00	−0.45	−0.01
Maple/beech/birch	–	−0.54	−0.18	–	−0.12
Aspen/birch	−0.16	−0.15	−0.29	−0.13	–
Alder/maple	–	–	–	−0.26	–
Western oak	–	–	–	−0.61	–
Tanoak/laurel	–	–	–	−0.10	–
Other hardwoods	–	−0.17	−1.92	0.04	−0.09
Woodland hardwoods	−0.45	–	–	−0.02	0.00
Tropical hardwoods	–	–	–	–	44.12
Exotic hardwoods	–	−14.45	–	−136.32	0.07
Nonstock	−0.12	−0.50	−0.19	−0.08	−0.01

A value of 1 indicates a ‘perfect’ fit, 0 suggests the model is no better than the mean, and negative values indicate a poor fit. Note that dashes (–) indicate no data for the forest type and region.

The general pattern of discontinuity was further confirmed by the equivalence tests for mean and median ratios between field- and model-based estimates of CWD C density. The mean of ratios in all cases were greater than the median of ratios. Only model-based estimates in the PNW had broad equivalence (±0.25, confidence limits of the ratios with 0.75 and 1.25) with field-based estimates (Table 3). The mean of ratios in the NE and North Central (NC) regions were close to the broad zone of indifference, although the data were too variable to be certain.

**Table 3:** Results of the equivalence tests comparing field- and model-based estimates of coarse woody debris carbon density (Mg•ha^−1^).

Region	Meanof ratios	Confidencelimits (90%)	Equival.	Medianof ratios	Confidencelimits (90%)	Equival.
**Public forest land**						
Northeast	0.728	(0.576–0.880)	ne	0.581	(0.607–0.723)	ne
North Central	1.753	(0.564–2.942)	ne	0.703	(0.607–0.826)	ne
South	1.100	(0.739–1.460)	ne	0.675	(0.562–0.807)	ne
Intermountain West	2.157	(1.629–2.685)	ne	1.496	(1.352–1.650)	ne
Pacific Northwest	2.507	(1.871–3.142)	ne	0.918	(0.893–0.945)	broad
**Private forest land**						
Northeast	1.072	(0.720–1.424)	ne	0.662	(0.487–0.684)	ne
North Central	0.901	(0.749–1.056)	ne	0.679	(0.613–0.756)	ne
South	4.606	(3.011–6.202)	ne	0.756	(0.702–0.818)	ne
Intermountain West	2.097	(1.562–2.633)	ne	1.983	(1.507–2.514)	ne
Pacific Northwest	4.496	(2.511–6.480)	ne	0.850	(0.816–0.884)	broad

Broad equivalence = 0.75 to 1.25, narrow equivalence = 0.90 to 1.10, and ne = no equivalence.

**Figure 5:**
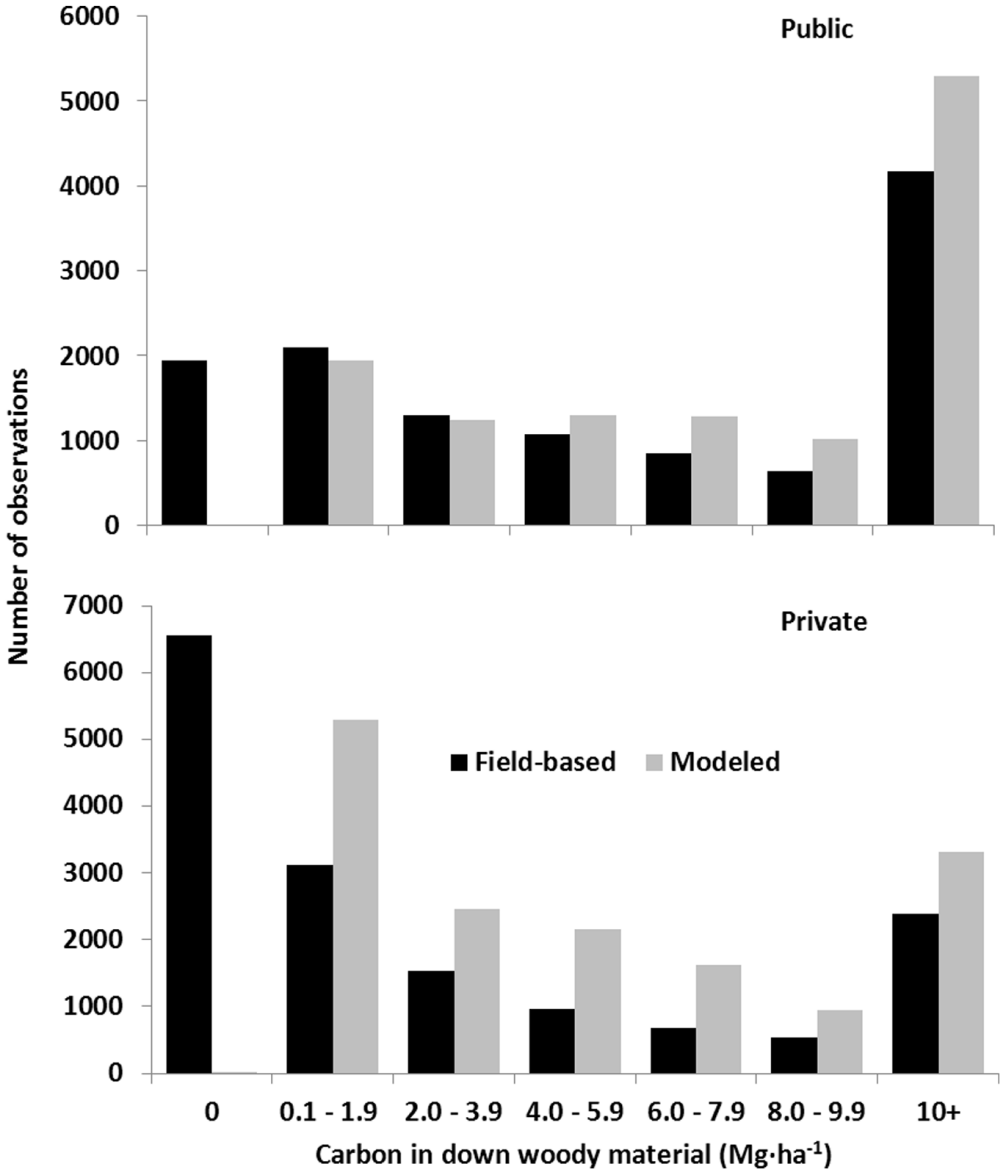
Distribution of observations by field- and model-based estimates of coarse woody debris C density. Model-based estimates of coarse woody debris carbon density are calculated using live tree carbon density. Observations without estimates of live tree carbon will not have an estimate of coarse woody debris carbon for pieces. However there may still be an estimate of coarse woody debris for the observations since the C density from slash piles is calculated using stand age and regional variables. Observations with no live tree carbon density and a stand age of zero will result in a total coarse woody debris (i.e. pieces and slash piles) estimate of zero.

To assess potential reasons for the large differences in model versus field-based CWD C density estimates, frequency of observations by C density classes on all plots in the study area were plotted against C density in CWD for field- and model-based estimates (Figure 5). Not surprisingly, very few (<0.05 percent) model-based estimates were zero, whereas no CWD was identified by the field-based inventory on 16 percent of public forest land and 41 percent of private forest land observations.

**Figure 6:**
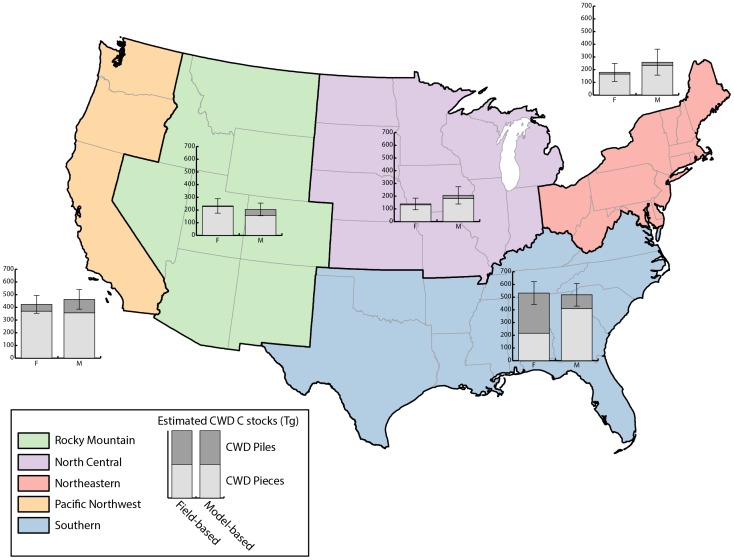
Regional estimates of coarse woody debris C stocks. Field- and model-based population estimates (with standard errors) of total coarse woody debris (i.e., pieces and slash piles) carbon stocks by region in the U.S. Population estimates include all observations in the study.

For all states in which CWD was sampled, the region- and forest type-specific CWD models (eq 1–4) overestimated the cumulative total CWD C density by 9 percent (145.1 Tg) within the NGHGI. The relatively small difference was caused by contrasting results for each CWD component. The model-based population estimate of C stocks from CWD pieces was 17 percent (230.3 Tg) greater than the field-based estimate, while the model-based estimate of C stocks from CWD slash piles was 27 percent (85.2 Tg) smaller than the field-based estimate. Although the disparity in model- and field-based population estimates of total CWD C stocks was less than 3 percent (13.5 Tg) in the SO region, it was small because a large overestimation for the model-based C in CWD pieces (47 percent; 194.5 Tg) was compensated for by a large underestimation for the model-based C in CWD piles (98 percent; 208.0 Tg) (Figure 6). The variation in differences by component led to large variations in differences between state-level model- and field-based population estimates of CWD C density (Table 4). Field-based estimates in Tennessee, for example, were more than 105 percent (35.5 Tg) larger than model-based estimates.

**Table 4:** Field- (*C_FLD_*) and model-based (*C_MOD_*) estimates of state-level C density in CWD (Tg) and the associated adjustment factor (*C_adj_*) used to amend estimates of CWD C density in the 2013 NGHGI report [24].

State	*C_FLD_*	*C_MOD_*	*C_adj_*	State	*C_FLD_*	*C_MOD_*	*C_adj_*
Alabama	58.65	48.46	1.21	Montana	82.42	62.70	1.31
Arizona	20.52	16.37	1.25	Nebraska	1.50	3.13	0.48
Arkansas	42.25	39.79	1.06	New Hampshire	7.61	14.00	0.54
California	97.03	139.60	0.70	New Jersey	0.85	5.02	0.17
Colorado	39.94	44.55	0.90	New York	36.06	52.88	0.68
Connecticut	4.48	5.25	0.85	North Carolina	29.30	46.61	0.63
Delaware	0.49	1.15	0.43	North Dakota	0.38	1.18	0.32
Florida	45.82	35.18	1.30	Ohio	16.03	22.32	0.72
Georgia	14.64	58.39	0.25	Oklahoma	22.20	19.26	1.15
Idaho	66.57	60.63	1.10	Oregon	150.16	173.87	0.86
Illinois	5.81	13.27	0.44	Pennsylvania	35.12	47.36	0.74
Indiana	10.07	13.59	0.74	Rhode Island	0.55	1.03	0.54
Iowa	5.70	7.02	0.81	South Carolina	15.22	32.55	0.47
Kansas	3.49	5.17	0.68	South Dakota	2.01	3.15	0.64
Kentucky	19.40	28.88	0.67	Tennessee	69.39	33.91	2.05
Louisiana	27.60	31.14	0.89	Texas	118.01	61.22	1.93
Maine	36.00	43.59	0.83	Utah	22.89	20.36	1.12
Maryland	3.28	8.27	0.40	Vermont	11.02	13.46	0.82
Massachusetts	3.75	9.07	0.41	Virginia	34.97	41.56	0.84
Michigan	34.51	50.36	0.69	Washington	174.86	148.51	1.18
Minnesota	34.46	35.41	0.97	West Virginia	23.60	35.25	0.67
Mississippi	34.73	43.21	0.80	Wisconsin	19.17	39.06	0.49
Missouri	21.74	35.27	0.62				

## Discussion

Recently compiled field-based estimates of CWD C density (i.e., per-unit-area) were significantly different from model-based estimates, with modeled estimates generally overestimating CWD C density on sites with small amounts of material and underestimating CWD C on sites with large amounts of material. The per-unit-area divergence was also evident at the state level but, collectively, the difference between methods for all states in the study was less than 9 percent. The relatively small absolute difference between model- and field-based estimates at the per-unit-area and population scales was not a result of good model fits, rather it reflected the models overestimating the contribution of C from CWD pieces and underestimating the contribution of C from CWD in slash piles. The largest divergence was found on private land in the SO and PNW regions, where the differences are driven by the inclusion of plot-specific empirical estimates, rather than modeled regional estimates of slash piles from forest management activity. In both regions, there were clear differences in stand attributes between public and private forest land, indicative of varying levels of forest management intensity. Stands on private forest land were markedly younger, less dense, and had substantially more CWD C in the form of slash than public forest lands. In fact, in the SO region where high intensity, even-aged management is common [40], the overwhelming majority of CWD was from slash piles. This result was less prominent in the PNW region where there was a large amount of CWD across ownerships but, given the size of forest biomass in the region, the absolute contribution of piles was substantial.

There was also substantial variation in the field-based estimates of CWD C on private forest land within the SO and PNW regions, which suggests there are also large differences in management and harvesting practices within private forest lands. The large differences between estimation approaches and variation in estimates may also be due, in part, to the sample design and population estimation procedures [22] used to scale per-unit-area estimates to the state-level population totals. Estimating the volume of CWD in slash piles is not exact and, depending on where the pile falls, it may end up producing a per-unit-area estimate that is not operationally realistic. The field-based population estimates from the arid IW region were 14 percent larger than modeled estimates in the region with piles only minimally contributing to CWD density, perhaps due to wildfires and fuel management in the region [41]. Field- and model-based estimates of CWD C density were most similar in the NE and NC regions – where slash piles were a relatively minor component of the CWD C estimate – for both ownerships. That said, the large variation in mean estimates was such that, despite good agreement between approaches, they were not statistically equivalent. The variation is evident when scaling to the state level, where field-based estimates of CWD C density were 33 percent smaller than model-based estimates in the NC region, and 31 percent smaller in the NE. The large variation in field-based estimates is likely driven by a combination of climatic factors (e.g., drought), natural disturbance (e.g., wind/ice storms), and forest management activities that are not fully accounted for in the model-based estimates [42], [43].

The differences in model- and field-based estimates of CWD C density may also be due to the indirect assumption that CWD is present on all sites, which is inherent in the ratio estimates used to model CWD from live tree C density. Similar findings for model- versus field-based estimates of standing dead tree C were found by Woodall et al. [44] echoing inherent limitations associated with modeling forest C pools. Only observations without an estimate of live tree C density (*n* = 484, 1.8 percent of all observations) would not have an associated estimate of C density for CWD pieces (note that per-unit-area estimates of C in CWD piles is a regional estimate that does not rely on live tree C density). While this assumption may be accurate for most forested sites, field-based estimates only reflect CWD measured along transects radiating from subplot centers. The field data revealed that more than 16 percent of observations on public forest land (*n* = 1,942) and 41 percent of observations on private forest land (*n* = 6,548) lacked measurement of any CWD. The absence of CWD in the field data may be due, in part, to measurement error (i.e., obscured CWD in dense understories) and/or sampling intensity as well as the high spatial variability of CWD. The disparity between model- and field-based estimates is further exacerbated by plots that have a small amount of live tree C, which results in model-based estimates that are inconsistent with the definition of the CWD population and/or ecologically untenable (0.001 Mg•ha^−1^). There is also the potential for extremely large model-based estimates resulting from FIA’s mapped plot conditions that are extremely small. Model-based estimates on these conditions, referred to as slivers (i.e., parts of FIA plots; [23]), like all other conditions are expanded by the size of the condition. Conditions with extremely small areal extent but with extremely large trees may result in per-unit-area estimates which are not biologically realistic, resulting in outliers.

Not only was there significant disagreement between model- and field-based estimates of CWD C density by region and ownership, there was also a general lack of fit between model estimates and forest type by region. With few exceptions (e.g., Douglas-fir in the IW region), the model-based estimates did not reflect the field-based CWD C density estimates, leading to large differences across spatial scales. Given the large variation in field-based estimates by forest type and region, it is not surprising the individual models performed poorly. There are many natural and anthropogenic factors driving CWD dynamics in forest ecosystems [3] and, while the relationship between live tree C density and CWD C provides a reasonable theoretical construct [16], it does not capture the variation in CWD C density across the U.S. analogous to results found by Woodall et al. [44] for standing dead tree C. The recent compilation of CWD C density information represents the latest effort to improve the accuracy and reliability of estimates of forest attributes in the FIA program [45]. The empirically based estimates not only provide an accurate assessment of the current downed dead wood resource in the U.S., they also represent the “first look” at a dataset that will continue to be utilized and expanded to assess trends in CWD dynamics in the face of global change.

The incorporation of the field-based estimates of CWD C density into the Land Use, Land Use Change, and Forestry (LULUCF) chapter of the U.S.’s 2013 NGHGI report represents the culmination of more than 10 years of research and development on downed and dead wood in the FIA program. Overall, the model-based approach that relied on live tree C density on plots overestimated CWD C density by approximately 9 percent based on the latest compilation (2010) of field-based estimates for the U.S. The relatively small difference in estimates does not reflect the large and statistically significant per-unit-area differences that, when expanded to the population, result in substantial state-level differences in CWD C density. The *C_adj_* ratio developed from the difference between model- and field-based CWD C density estimates can be used in the 2013 NGHGI report to adjust all previous estimates of downed dead wood dating back to the baseline year, 1990 [24]. Since previous NGHGI reports relied solely on model-based estimates of downed dead wood C density, comparing adjusted estimates in the 2013 report with past reports would not be appropriate.

A future refinement would be to revisit the alignment between the C pool definitions of DWM and the forest floor. Currently, FIA’s inventory of FWD (transect diameter <7.62 cm) is not included in the field-based estimates of dead wood in the NGHGI [24], as it is currently a modeled estimate included in the forest floor pool. There are two approaches to resolving this dilemma. First, the forest floor model could be revised to incorporate the field-based estimates of all sizes of FWD. Given the size of FWD C stocks seen across the U.S., the dynamics of DWM C stocks might be better monitored when all dead wood is included. A second approach would be to redefine the DWM pool to include medium and large FWD (transect diameter >0.64 cm and <7.62 cm) so that the near entirety of FIA’s DWM inventory could inform the monitoring of DWM C pools across the U.S. As FIA’s soil monitoring program includes small FWD (transect diameter <0.64 cm) as part of their forest floor sampling protocols [28], this approach may engender greater alignment between both the field inventory components (e.g., dead wood inventory databases directly aligned with NGHGI C pools) and C dynamics research (e.g., dead wood pool containing nearly all dead wood components before decomposition into soil horizons).

Finally, the incorporation of field-based estimates of CWD C density into the 2013 NGHGI report is part of a larger effort to align estimates of forest C available in the publically available FIA DataMart (http://fiatools.fs.fed.us; [46]) with those in the NGHGI report [18], [19], [44], [47]. It is also hoped that the recent release of the plot-level data and associated compilations will facilitate research on CWD dynamics in the U.S. and further verify the importance of this component in forest C reporting. As an addendum, the data should also prove useful in bioenergy assessments where a renewed interest in forest-derived biomass for energy has created demand for material historically left on harvest sites [48]. Until the recent compilation of CWD in the FIA program, industry, public land managers, and policymakers have relied on model-based estimates which may have significantly over- or underestimated the CWD C/biomass on forest land. The field-based CWD biomass/C estimates reduce uncertainty and thus improve our understanding of the dead wood resource for wildlife habitat [1], provide a more accurate reflection of potential feedstocks for renewable energy projects [48], [49], and enhance our ability to track changes in forest C attributes following forest management activities and climate change events [42], [43]. The plot- and population-level estimates should also inform forest fire management applications and fuel reduction efforts across multiple spatial scales [50].

### Conclusions

Coarse woody debris C density is not entirely a function of a forest stand’s live tree C stocks. Ownership, disturbance history, and a host of biotic/abiotic factors greatly complicate the relationship between live and dead wood C stocks. Such models may provide a “plausible” CWD C population total at the national scale but potentially biased estimates by CWD component and at the plot level. Models will almost always predict CWD C stocks whenever there is appreciable live tree C, but greatly underestimate CWD C stocks when mortality events (whether stochastic or management related) result in low live tree C stocks and commensurately large CWD C stocks. Basing CWD C stocks on a field-based inventory will not only improve NGHGIs, but also enable accurate monitoring of CWD resources at small scales and as affected by potential management and climate change events.

## References

[1] Maser C, Anderson RG, Cromack K Jr, Williams JT, Martin RE (1979) Dead and down woody material. In: Thomas JW editor. Wildlife habitats in managed forests - the Blue Mountains of Oregon and Washington. USDA Agricultural Handbook 553, USDA Forest Service, Washington DC. 78–95.

[2] Franklin JF, Shugart HH, Harmon ME (1987) Tree death as an ecological process. BioScience 37: 550–556.

[3] Harmon ME, Franklin JF, Swanson FJ, Sollins P, Gregory SV, et al.. (1986) Ecology of coarse woody debris in temperate ecosystems. In Macfadyen A, Ford ED, editors. Advances in Ecological Research Volume 15(133). Academic Press Inc., London.

[4] Spetich MA, Shifley SR, Parker GR (1999) Regional distribution and dynamics of coarse woody debris in Midwestern old-growth forests. Forest Science 45: 302–313.

[5] Arseneault J, Fenton NJ, Bergeron Y (2012) Effects of variable canopy retention harvest on epixylic bryophytes in boreal black spruce – feathermoss forests. Canadian Journal of Forest Research 42: 1467–1476.

[6] Robert E, Brais S, Harvey BD, Greene D (2012) Seedling establishment and survival on decaying logs in boreal mixedwood stands following a mast year. Canadian Journal of Forest Research 42: 1446–1455.

[7] Hagan JM, Grove SL (1999) Coarse woody debris: humans and nature competing for trees. Journal of Forestry 97: 6–11.

[8] Rollins MG, Keane RE, Parsons RA (2004) Mapping fuels and fire regimes using remote sensing, ecosystem simulation, and gradient modeling. Ecological Applications 14: 75–95.

[9] Bigler C, Veblen TT (2011) Changes in litter and dead wood loads following tree death beneath subalpine conifer species in northern Colorado. Canadian Journal of Forest Research 41: 331–340.

[10] Bull EL, Parks CG, Torgersen TR (1997) Trees and logs important to wildlife in the Interior Columbia River Basin. USDA Forest Service General Technical Report PNW-GTR-391. Pacific Northwest Research Station, Portland, OR.

[11] Marcot BG, Ohmann JL, Mellen-McLean K, Waddell KL (2010) Synthesis of regional wildlife and vegetation field studies to guide management of standing and down dead trees. Forest Science 56: 391–404.

[12] Chojnacky DC, Heath LS (2002) Estimating down deadwood from FIA forest inventory variables in Maine. Environmental Pollution 116: 25–30. 10.1016/s0269-7491(01)00243-3 11833911

[13] Bradford J, Weishampel P, Smith M-L, Kolka R, Birdsey R, et al (2009) Detrital carbon pools in temperate forests: magnitude and potential for landscape-scale assessment. Canadian Journal of Forest Research 39: 802–813.

[14] Hoover CM, Leak WB, Keel BG (2012) Benchmark carbon stocks from old-growth forests in northern New England, USA. Forest Ecology and Management 266: 108–114.

[15] Smith JE, Heath LS, Woodbury PB (2004) How to estimate forest carbon for large areas from inventory data. Journal of Forestry 102: 25–31.

[16] Smith JE, Heath LS, Skog KE, Birdsey RA (2006) Methods for calculating forest ecosystem and harvested carbon with standard estimates for forest types of the United States. USDA Forest Service General Technical Report NE-343. Northeastern Research Station. Newtown Square, PA.

[17] Woodall CW, Heath LS, Smith JE (2008) National inventories of dead and downed forest carbon stocks in the United States: Opportunities and Challenges. Forest Ecology and Management. 256: 221–228.

[18] Woodall CW, Heath LS, Domke GM, Nichols MC (2011) Methods and equations for estimating aboveground volume, biomass, and carbon for trees in the U.S. forest inventory, 2010. USDA Forest Service General Technical Report NRS-88. Northern Research Station, Newtown Square, PA.

[19] Domke GM, Woodall CW, Smith JE (2011) Accounting for density reduction and structural loss in standing dead trees: Implications for forest biomass and carbon stock estimates in the United States. Carbon Balance and Management 6: 14. 22115425 10.1186/1750-0680-6-14 PMC3283479

[20] United States Environmental Protection Agency (2012) Forest sections of the Land Use, Land Use Change, and Forestry chapter, and Annex. In: US Environmental Protection Agency, Inventory of US Greenhouse Gas Emissions and Sinks: 1990–2010. EPA 430-R-12–001. Available: http://www.epa.gov/climatechange/emissions/usinventoryreport.html. Accessed 8 January 2013.

[21] Woodall CW, Rondeux J, Verkerk P, Stahl G (2009) Estimating dead wood during national inventories: A review of inventory methodologies and suggestions for harmonization. Environmental Management 44: 624–631. 19701595 10.1007/s00267-009-9358-9

[22] Woodall CW, Monleon VJ (2008) Sampling, estimation, and analysis procedures for the Down Woody Materials indicator. USDA Forest Service General Technical Report NRS-22. Northern Research Station, Newtown Square, PA.

[23] Bechtold WA, Patterson PJ (2005) The enhanced Forest Inventory and Analysis program–national sampling design and estimation procedures. USDA Forest Service General Technical Report SRS-80. Southern Research Station, Asheville, NC.

[24] United States Environmental Protection Agency (2013) Forest sections of the Land Use, Land Use Change, and Forestry chapter, and Annex. In: US Environmental Protection Agency, Inventory of US Greenhouse Gas Emissions and Sinks: 1990–2011. In press.

[25] Forest Carbon Portal (2012) Forest carbon project inventory. Available: http://www.forestcarbonportal.com/projects. Accessed: 26 November 2012.

[26] Intergovernmental Panel on Climate Change (2003) Good Practice Guidance for Land Use, Land-Use Change and Forestry. Available: http://www.ipcc-nggip.iges.or.jp/public/gpglulucf/gpglulucf_contents.html. Accessed: 23 January 2013.

[27] Westfall JA, Woodall CW, Hatfield MA (2013) A statistical power analysis of woody carbon flux from forest inventory data. Climatic Change In press.

[28] Woodall CW, Perry CH, Westfall JA (2012a) An empirical assessment of forest floor carbon stock components across the United States. Forest Ecology and Management 269: 1–9.

[29] Hardy CC (1996) Guidelines for estimating volume, biomass, and smoke production for piled slash. USDA Forest Service General Technical Report PNW-364. Pacific Northwest Research Station, Portland, OR.

[30] Woodall C, Conkling BL, Amacher MC, Coulston JW, Jovan S, et al.. (2010) The forest inventory and analysis database version 4.0: database description and users manual for phase 3. USDA Forest Service General Technical Report NRS-61. Northern Research Station, Newtown Square, PA.

[31] Woudenberg SW, Conkling BL, O'Connell BM, LaPoint EB, Turner JA, et al.. (2010) The Forest Inventory and Analysis Database: Database description and users manual version 4.0 for Phase 2. USDA Forest Service General Technical Report RMRS-GTR-245. Rocky Mountain Research Station, Fort Collins, CO.

[32] Hatfield MA (2010) Post-stratified estimation of coarse woody debris volume using the down woody materials sample of forest inventory and analysis. Thesis. University of Minnesota, St. Paul, MN.

[33] Harmon ME, Woodall CW, Fasth B, Sexton J (2008) Woody detritus density and density reduction factors for tree species in the United States: a synthesis. General Technical Report NRS-29. USDA Forest Service, Northern Research Station. Newtown Square, PA.

[34] Heath LS, Nichols MC, Smith JE, Mills JR (2010) FORCARB2: an updated version of the US Forest Carbon Budget Model. USDA Forest Service General Technical Report NRS-67. Northern Research Station, Newtown Square, PA.

[35] Vanclay JK, Skovsgaard JP (1997) Evaluating forest growth models. Ecological Modelling 98: 1–12.

[36] Robinson AP, Froese RE (2004) Model validation using equivalence tests. Ecological Modelling 176: 349–358.

[37] Robinson AP, Duursma RA, Marshall JD (2005) A regression-based equivalence test for model validation: shifting the burden of proof. Tree Physiology 25: 903–913. 15870057 10.1093/treephys/25.7.903

[38] Zhou XH, Dinh P (2005) Nonparametric confidence intervals for the one- and two-sample problems. Biostatistics 6: 187–200. 15772099 10.1093/biostatistics/kxi002

[39] R Development Core Team (2011) R: A Language and Environment for Statistical Computing. R Foundation for Statistical Computing, Vienna, Austria. http://www.R-project.org/.

[40] Wear DN, Greis JG (2002) Southern forest resource assessment: summary of findings. Journal of Forestry 100: 6–14.

[41] Covington WW, Sackett SS (1984) The effect of a prescribed burn in southwestern ponderosa pine on organic matter and nutrients in woody debris and forest floor. Forest Science 30: 183–192.

[42] Everham EM, Brokaw NVL (1996) Forest damage and recovery from catastrophic wind. The Botanical Review 62: 113–185.

[43] Fraver S, Wagner RG, Day M (2002) Dynamics of coarse woody debris following gap harvesting in the Acadian forest of central Maine, U.S.A. Canadian Journal of Forest Research. 32: 2094–2105.

[44] Woodall CW, Domke GM, Macfarlane DW, Oswalt CM (2012b) Comparing field-and model-based standing dead tree carbon stock estimates across forests of the US. Forestry 85: 125–133.

[45] Woodall CW (2012) Where did the U.S. forest biomass/carbon go? Journal of Forestry 110: 113–114.

[46] USDA Forest Service (2013) Forest inventory and analysis national program – data and tools - FIA data mart, FIADB Version 5.1. USDA Forest Service. Available: http://apps.fs.fed.us/fiadb-downloads/datamart.html. Accessed 23 January 2013.

[47] Domke GM, Woodall CW, Smith JE, Westfall JA, McRoberts RE (2012a) Consequences of alternative tree-level biomass estimation procedures on US forest carbon stock estimates. Forest Ecology and Management 270: 108–116.

[48] Domke GM, Becker DR, D’Amato AW, Ek AR, Woodall CW (2012b) Carbon emissions associated with the procurement and utilization of forest harvest residues for energy northern Minnesota, USA. Biomass and Bioenergy 36: 141–150.

[49] Sample A (2007) Bioenergy markets: new capital infusion for sustainable forest management? Pinchot Letter 11: 1–7.

[50] Stephens SL, McIver JD, Boerner REJ, Fettig CJ, Fontaine JB, et al (2012) The effects of forest fuel-reduction treatments in the United States. BioScience 62: 549–560.

